# Hippocampal Neurotransmitter Inhibition Suppressed During Gaming Explained by Skill Rather Than Gamer Status

**DOI:** 10.3389/fnhum.2020.585764

**Published:** 2020-12-07

**Authors:** Kelsey Prena, Hu Cheng, Sharlene D. Newman

**Affiliations:** ^1^Emerging Media Studies, College of Communication, Boston University, Boston, MA, United States; ^2^Psychological and Brain Sciences, College of Arts and Sciences, Indiana University, Bloomington, IN, United States; ^3^Department of Psychology, College of Arts and Sciences, University of Alabama, Tuscaloosa, AL, United States

**Keywords:** spatial memory, goal-directed spatial decision making, neurotransmitters, γ-aminobutyric acid, hippocampus

## Abstract

Goal-directed spatial decision making video games combine spatial mapping, memory, and reward; all of which can involve hippocampal excitation through suppression of an inhibitory neurotransmitter, γ-aminobutyric acid (GABA). In this study, GABA was measured before and after 30 min of video game play within a voxel around the hippocampus. It was predicted that all participants would experience a decrease in GABA during gaming as a result of in-game rewards; and, those who were most competitive with the goal-directed spatial decision making game would display lower hippocampal GABA concentrations after gaming. Those who were not competitive, because they were too skilled or not skilled enough, would demonstrate higher hippocampal GABA concentrations after gaming. While there were no significant differences in hippocampal GABA before and after gaming for gamers and non-gamers alike, there was a significant quadratic regression between performance on a spatial working memory task and post-gaming hippocampal GABA concentrations.

## Introduction

The hippocampus is the region of the brain responsible for forming declarative (facts, associations) and spatial memory (locations; see Squire et al., 1993). Both processes are positively associated with hippocampal volume (Petersen et al., 2000; Nedelska et al., 2012, respectively). Interestingly, video gameplay has also been positively associated with hippocampal volume (Gleich et al., 2017) and hippocampal-dependent declarative memory performance (Prena et al., 2018b).

A possible explanation for the relationship between video gameplay and hippocampal activity is a process called reward anticipation. When sensory input from the surrounding environment indicates reward potential, the activity at the hippocampus is facilitated by suppressing its dominant inhibitory neurotransmitter, γ-aminobutyric acid (GABA; see Luo et al., 2011; Blum et al., 2012; Gasbarri and Pompili, 2014). GABA suppression caused by anticipatory reward facilitates hippocampal activation to promote spatial learning (Moser and Moser, 1998; Li et al., 2003) and declarative memory (Adcock et al., 2006; de Lima et al., 2006; Ostrovskaya et al., 2014). Alternatively, increases in GABA concentrations in the hippocampus preserve synaptic connections (Ostrovskaya et al., 2014; Shen et al., 2017) and suppress unwanted memories (Schmitz et al., 2017). Overproduction of GABA in the hippocampus has been associated with memory deficits often displayed in people with Alzheimer’s disease or Down syndrome (Kleschevnikov et al., 2004; Ambrée et al., 2009; Chakrabarti et al., 2010). In this experiment, we explore the relationship between hippocampal GABA, spatial memory performance, and video gameplay with the hope that it might reveal information for how video games alter hippocampal GABA concentrations.

### Goal-Directed Spatial Decision Making

Video game mechanics, or rules about how players interact within a video game (see Boyan and Sherry, 2011), can have important effects on how players experience the game. Mechanics can be rewarding (i.e., overcoming an opponent in battle, earning game currency, or unlocking access to locations), or punishing (i.e., loss of resources, loss of avatar’s energy, or loss of game progression) and, they can also lead to an understanding of the worth of achievements, facilitate skill development, and provide a context for learning, practicing, and mastering the skills necessary to complete the game (Juul, 2009; see Juul, 2010; see King et al., 2010). Video game mechanics can activate or suppress different regions of the brain based on the types of tasks players are asked to complete.

A processing demand that could account for reward-related hippocampal neurotransmitter changes during video gaming is goal-directed spatial decision making. Goal-directed spatial decision making is deciding how to behave and navigate within a three-dimensional environment in pursuit of a reward (Viard et al., 2011). This demand combines reward anticipation (Adcock et al., 2006), novel information processing (Wittmann et al., 2007), and spatial learning of the environment (see Squire et al., 1993; for GABA inhibition see Cui et al., 2008); all of which trigger hippocampal activation. It is unclear what effect video gaming will have on hippocampal GABA because this particular relationship is novel in research. But, research suggests that goal-directed spatial decision making within virtual worlds can increase hippocampal activity in humans (Cornwell et al., 2008; Viard et al., 2011; Clemson and Stark, 2015). Hippocampal activity (blood-oxygenation level-dependent, BOLD, response) has been associated with how close participants are to a known goal within a virtual maze (Viard et al., 2011); and, hippocampal activity increased as participants approached the goal. Similarly, theta oscillations in a magnetoencephalographic (MEG) system revealed hippocampal activation as people learned how to navigate to hidden platforms and predict more efficient paths between two locations (Cornwell et al., 2008). Furthermore, goal-directed behavioral learning in mice can be interrupted when hippocampal GABA is increased through a pharmacological GABA agonist (Le Merre et al., 2018).

Video games have the potential to be a good medium for observing goal-directed spatial decision making effects on hippocampal activity. This is because video games can establish a navigable world with clear reward systems using game mechanics. Video games can require players to memorize routes, series of actions, the timing of those actions, and perceptual cues that inform players of reward potential. Two months of game-training on the goal-directed spatial decision making game Super Mario 64 led to heightened hippocampal response when observing game-related scenes (BOLD response; Gleich et al., 2017). This demonstrates training-related changes that were observed after gaming ended. Furthermore, earlier research using this current dataset indicated that gamers (gaming for at least 5 h weekly) had significantly lower GABA concentrations than nongamers (gaming no more than 1 h weekly; Prena et al., 2018a).

### Challenge

Important triggers for decreasing hippocampal GABA are anticipatory reward and spatial mapping, both of which are experienced during goal-directed spatial decision making (Viard et al., 2011). The hippocampus is most excited when the reward is seen as attainable but not guaranteed (reward is achieved around 50%, as opposed to 0% or 100%; Hollerman and Schultz, 1998; Dreher et al., 2005). This idea has been previously applied to video games through flow theory (Csikszentmihalyi, 1990). The gameplay is intensified when the player perceives their skill level as equal to the challenge the video game presents (see Sherry, 2004). When participants cannot learn to predict the achievement of rewards or avoidance of punishments within the game (nearing 0%) they will grow frustrated. When participants achieve rewards and avoid punishments too easily (nearing 100%), they grow bored of the game. It is expected that those who compete with the game (rather than fail or dominate within it) will experience the greatest decrease in hippocampal GABA concentrations. To explore the relationship between hippocampal GABA concentrations and goal-directed spatial decision making in video gaming, it was hypothesized that:

H1: there will be an overall decrease in hippocampal GABA concentrations from before gaming to after gaming.

It was also expected that spatial memory would influence GABA concentrations during gaming quadratically. Those with spatial memory skill levels most competitive with game demands will experience the greatest reward during gaming. It was hypothesized that:

H2: post-gaming hippocampal GABA will be quadratically related to the level achieved within the goal-directed spatial decision making game.

H3: spatial working memory performance will be quadratically related to post-gaming GABA concentrations.

## Materials and Methods

### Participants

Data from 37 male participants (age 18–26) are presented in this experiment. They were recruited as self-identified non-gamers who played video games for under 1 h a week (*n* = 17) or gamers group who played at least five a week (*n* = 20). Participants were screened for safe entry into the magnetic resonance image (MRI) scanner *via* email before they were allowed to schedule time in the lab for the behavioral and imaging tasks. Originally, 57 participants were recruited for this study, but issues with excessive participant motion or inadequate shimming lead to poor magnetic resonance spectroscopy (MRS) data quality and unusable results. All participants gave written informed consent as approved by the university’s Institutional Review Board.

### Procedure

Upon entering the laboratory, participants re-completed the MRI screening form and completed informed consent forms. They performed working memory and spatial memory tasks, as well as other cognitive batteries outside the scope of this report (selective attention and inhibition). Participants completed a questionnaire about gaming habits and were then led to the scanning facility. They underwent a high-resolution anatomical scan, during which they were instructed to lie still. We conducted a single voxel MRS scan, and then participants played a video game for 30 min in the scanner. After 30 min, they were again instructed to lie still and underwent a final MRS scan, and then alerted that participation was over. They were removed from the scanner, compensated at $10/h ($25.00 total), and provided a three-dimensional copy of the brain scan.

The game selected was Sonic Adventure 2, a platform game where players respond to obstacles and overcome enemies, and coins, treasures, and goals incentivize navigation. It is important to acknowledge that this game is not a two-dimensional side-scrolling game where navigation is limited to right and left, up and down, like some platform games. Rather, the game is three-dimensional (including a three-dimensional map) and uses a third-person point of view. Players can navigate in all different directions on the map. Failure results in setbacks to earlier locations in the game (either the start of the level or a checkpoint). Players must then use spatial memory to remember paths taken, item/enemy locations, and decisions made to arrive back at and advance past locations where they experienced failures.

### Variables

#### Working Memory

A digital version of the digit span task (Baddeley and Hitch, 1974; see Baddeley, 2003) was presented using the Psychological Experiment Building Language (PEBL). A sequence of digits was presented on a computer screen, and participants were asked to repeat the sequence, in reverse order from which they were revealed. The sequence increased by one digit after every two trials. Participants were allowed one error, and after a second error, the task was terminated. Scores were calculated as the number of digits successfully remembered, *M* = 7.08, *SD* = 2.84.

#### Spatial Memory

Spatial working memory was assessed using the Corsi block-tapping test (Corsi, 1973) implemented in PEBL. Participants were shown nine stationary blue squares on a black screen and remembered the reverse order from which the squares brightened on the screen. Two trials were presented for each span; the sequence increased by one after two trials for that span were completed; once two sequential errors were made the task was terminated. Scoring was calculated as the number of squares in a row they could remember successfully, *M* = 5.97, *SD* = 0.77.

#### Time Spent Gaming Weekly

A modified version of the video game questionnaire to measure time spent gaming weekly (Anderson and Dill, 2000) was administered. Participants reported hours spent gaming for each day of the week in an average week over 6 months before participation. Days of the week were separated into three segments to aid in recall (e.g., before class, between classes but before dinner, after dinner). The final measure was calculated as the sum of the hours spent gaming in each of the segments, *M* = 11.14, *SD* = 14.51. Participants also listed the games that they play and provided how frequently they played those games.

#### Familiarity With Sonic Adventure 2

Participants were asked if they had experience playing Sonic Adventure 2 and responded with “none” (*n* = 31), “yes, a little” (*n* = 6), or “yes, a lot” (*n* = 0). None of the participants responded with “yes, a lot,” resulting in a dichotomous variable.

#### Sonic Level

The map within the video game has distinct checkpoints and levels. These accomplishments are made obvious by a change of the task at hand and changes to the landscape and environment. The Sonic level was scored by passing particular checkpoints and levels within the game. In most cases, this measure coincided with clear levels identified within the game. However, some longer levels were broken down into smaller sections through checkpoints. These checkpoints were also counted towards the number of Sonic levels achieved (*M* = 3.24, *SD* = 1.50, range = 6).

#### MRI Data Acquisition

A 3 Tesla Siemens Prisma scanner located in the university’s imaging facility was used. The T1-weighted high-resolution anatomical image was acquired. The voxel used to acquire MRS data was placed in the right hippocampus (voxel size 40 × 17 × 17 mm^3^). The MEGA-PRESS J-editing sequence was used for GABA measurement: TR/TE = 1,500/30 ms, bandwidth = 1,000 Hz, 512 data points, number of measurements = 128. See Supplementary Figure 1 in Supplementary Materials for details regarding acquisition parameters and MRS voxel placement.

LCModel 6.3-0L (Provencher, 2018) was used to fit the difference spectrum as a weighted linear combination of a basis set provided by Dydak and Murdoch (2017). This basis was generated from density matrix simulations of the sequence using published values for chemical shifts and J-couplings from and Kaiser et al. (2008). The basis set contains GABA, glutamate/glutamine complex, and N-acetyl-aspartate (NAA). These metabolites are reported in institutional units. The “off” spectrum is a standard MRS spectrum and can be analyzed using standard procedures in LCModel to quantify NAA, creatine, and other metabolites. A nonlinear baseline was incorporated in both fittings to account for artifacts such as the highly variable lipid and macromolecule signals. By combining the results of the difference spectrum and “off” spectrum, the ratio of GABA/Cr was obtained. The Cramér-Rao lower bounds (CRLB) estimated as the relative standard deviation for each fitted component were also calculated using LCModel. Only fitting results with CRLB values <20% were used for further statistical analysis with initial GABA concentrations, *M* = 0.10, *SD* = 0.04, and GABA after video gaming, *M* = 0.10, *SD* = 0.04.

## Results

Participants were an average age of 21.08 years old. Nongamers reported an average of 0.58 h of weekly gaming, *SD* = 0.59, and gamers reported an average of 19.38 h of weekly gaming, *SD* = 14.65. This difference was significant, *t*_(19.08)_ = 5.48, *p* > 0.001. Additional two-tailed independent samples *t*-tests demonstrated that familiarity with Sonic Adventure 2 was not a significant predictor of GABA before *t*_(35)_ = 1.07, *p* = 0.098, or after gaming *t*_(35)_ = 1.89, *p* = 0.067. Table 1 displays the means, standard deviations, and confidence intervals of our variables of interest, as a whole and divided by video game status.

**Table 1:** Descriptive statistics for variables.

Key variables	Non-gamers (*n* = 17)			Gamers (*n* = 20)			Total (*n* = 37)		
	M	SD	95% CI	M	SD	95% CI	M	SD	95% CI
Age	21.76	2.17	(20.73, 22.79)	20.45	1.73	(19.69, 21.21)	21.05	2.03	(20.40, 21.70)
Corsi scores	5.88	0.80	(5.50, 6.26)	6.05	0.76	(5.72, 6.38)	5.97	0.77	(5.72, 6.22)
Digit-span scores	7.35	3.30	(5.78, 8.92)	6.85	2.60	(5.71, 7.99)	7.08	2.91	(6.14, 8.02)
Pre-gaming GABA	0.11	0.04	(0.09, 0.13)	0.08	0.03	(0.07, 0.09)	0.10	0.04	(0.09, 0.11)
Post-Gaming GABA	0.10	0.04	(0.08, 0.12)	0.09	0.04	(0.07, 0.11)	0.10	0.04	(0.09, 0.11)
Sonic level	2.59	0.80	(2.21 ,2.97)	3.84	1.77	(3.06, 4.62)	3.25	1.52	(2.76, 3.74)
Familiarity with sonic	*n* = 2	-	-	*n* = 4	-	-	*n* = 6	-	-
Weekly gaming	0.59	0.59	(0.31, 0.87)	20.10	14.64	(13.68, 26.52)	11.14	14.51	(6.56, 15.82)

### H1

The first hypothesis predicting that hippocampal GABA concentrations will be lower after gaming was not supported. A 2 (Time: before gaming or after gaming) × 2 (Gamer Status: gamer or nongamer) mixed-subjects ANOVA was used to compare pre-and post-gaming hippocampal GABA concentrations between gamers and non-gamers. There was not a significant main effect for time, *F*_(1,35)_ = 0.10, *p* = 0.759, partial *η*^2^ = 2.67E-3, or gamer status *F*_(1,35)_ = 3.26, *p* = 0.079, partial *η*^2^ = 0.08. The interaction effect between time and gamer status for hippocampal GABA concentrations was also not significant, *F*_(1,35)_ = 2.21, *p* = 0.146, partial *η*^2^ = 0.05. See Table 1 for descriptions of each group.

### H2

**Figure 1:**
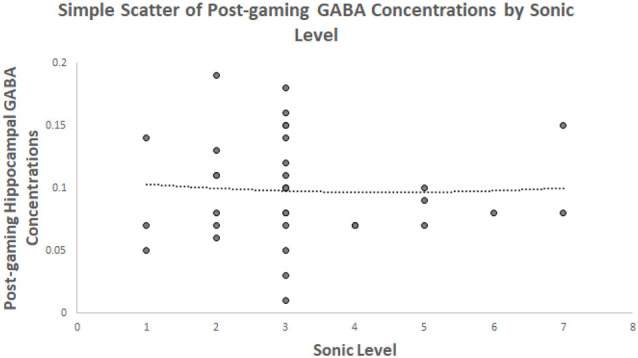
Depicts the relationship between hippocampal γ-aminobutyric acid (GABA) concentrations and the level of Sonic game play achieved showing a quadratic trendline.

The second hypothesis predicted that the level achieved will be quadratically related to post-gaming hippocampal GABA within the goal-directed spatial decision making game. It was not supported. A scatterplot between GABA and Sonic level is presented in Figure 1. A quadratic term was created for the level achieved in Sonic Adventure 2. Then, the linear and quadratic terms were entered in a regression. The model was not significant *F*_(2,36)_ = 0.02, *p* = 0.983, *r^2^* = 0.06. The level achieved was not a significant predictor of post-gaming hippocampal GABA concentrations using the linear term, *β* = −0.13, *t* = −0.17, *p* = 0.866. The quadratic term for level achieved was not a significant predictor of GABA either, *β* = 0.11, *t* = −0.15, *p* = 0.882.

### H3

**Figure 2:**
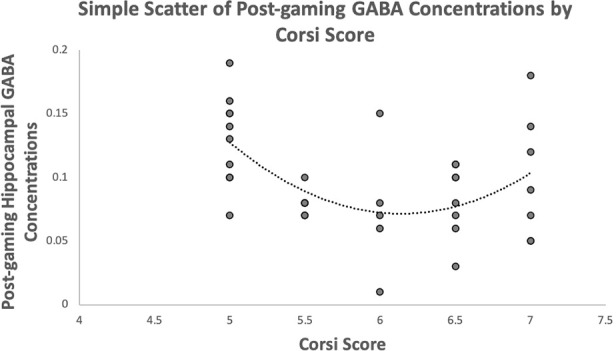
Depicts the relationship between hippocampal GABA concentrations and Corsi scores with a quadratic trendline.

The third hypothesis predicted that spatial working memory performance will be quadratically related to post-gaming GABA concentrations. This hypothesis was supported. A scatterplot of these variables is presented in Figure 2. The quadratic term for spatial working memory was created and then added to a regression model with the linear term. The model was significant, *F*_(2,36)_ = 6.84, *p* = 0.003, *r^2^* = 0.287. The linear term was significant, *β* = −9.94, *t* = −3.11, *p* = 0.004. The quadratic term was also significant, *β* = 9.64, *t* = 3.02, *p* = 0.005, suggesting that there is a quadratic relationship between spatial working memory and post-gaming GABA concentrations.

A *post hoc* quadratic regression demonstrated that spatial memory was not a significant predictor of pre-gaming hippocampal GABA concentrations, *F*_(2,36)_ = 1.69, *p* = 0.200, adjusted *r^2^* = 0.04. Neither the linear term, *β* = −6.56, *t* = −1.82, *p* = 0.078, nor the quadratic term, *β* = 6.52, *t* = 1.81, *p* = 0.080, were significant. Furthermore, a second regression demonstrated that neither the linear term for working memory, *β* = −0.35, *t* = −0.50, *p* = 0.624, nor the quadratic term, *β* = 0.30, *t* = 0.43, *p* = 0.669, could significantly predict post-gaming hippocampal GABA concentrations, *F*_(2,36)_ = 0.14, *p* = 0.868, adjusted *r^2^* = −0.05.

## Discussion

Using support from the flow theory, we predicted that those who were competitive with the spatial memory demands (as opposed to those who were too good or not good enough) would find the game the most rewarding. This would result in lower hippocampal GABA concentrations for those competitive with the game. Too much or too little skill would cause boredom or frustration, respectively, and result in higher hippocampal GABA concentrations. This was supported by the data. Performance on the spatial memory task-related quadratically to post-gaming hippocampal GABA concentrations. This also reflects conclusions drawn about hippocampal activation when anticipatory reward results in reward obtainment intermittently (Hollerman and Schultz, 1998; Dreher et al., 2005). Those who experienced reward intermittently, rather than always (too skilled) or never (not skilled enough), experienced lower hippocampal GABA concentrations.

The level achieved was not a significant predictor of post-gaming hippocampal GABA concentrations. This might suggest that a competitive interaction within the game (competition between player and skill) is more important than the number of in-game achievements. We had also expected that there would be an overall decrease in hippocampal GABA before and after gaming. However, the result of the third hypothesis (H3) demonstrates why this overall decrease did not occur: only those who were most competitive with the game were experienced reward. There was not a difference between gamers and non-gamers between pre-gaming and post-gaming GABA concentrations. Again, this makes sense after looking at the results indicating that competition between the skill and game predicted lower post-gaming hippocampal GABA concentrations.

The possibility that those who are better at spatial working memory have less hippocampal GABA was ruled out because initial GABA concentrations did not associate with spatial working memory performance linearly or quadratically. Furthermore, working memory without the spatial component could not predict post-gaming hippocampal GABA concentrations. This suggests that the spatial component of the Corsi task was important for predicting hippocampal GABA concentrations after gaming with a goal-directed spatial decision making video game; and this aligns with research indicating the importance of the hippocampus for spatial memory (for review see Squire et al., 1993).

Limitations of this study include the use of only one video game stimulus and a small homogenous sample. The small sample, along with the variance in spatial ability, may account for the null finding for hypothesis 1. *Post hoc*, exploratory analyses are presented in the Supplemental Material demonstrates that the high ability and not gamer status may lead to decreases in GABA after gameplay. Future work with a larger sample is necessary. No scanning data were collected while participants were gaming, so results might not reflect the all of changes that occurred during gaming. Future research should consider comparing other games that use goal-directed spatial decision making demands to games that do not use these demands. Future studies could also correlate hippocampal activity with the events occurring within the video game; and, other biological markers of flow, frustration, and boredom, should be compared to hippocampal activity, accounting for game-specific demands. This study is one of the first to examine hippocampal GABA concentrations in humans before and after gaming. While research on the topic is still novel, the current study demonstrates an important need to continue looking at the relationship between neurological activity and video game demands.

## Data Availability Statement

The raw data supporting the conclusions of this article will be made available by the authors, without undue reservation.

## Ethics Statement

The studies involving human participants were reviewed and approved by Institutional Review Board at Indiana University. The patients/participants provided their written informed consent to participate in this study.

## Author Contributions

All authors listed have made a substantial, direct and intellectual contribution to the work, and approved it for publication.

## Conflict of Interest

The authors declare that the research was conducted in the absence of any commercial or financial relationships that could be construed as a potential conflict of interest.
