# Supplementary material for: Hippocampal Neurotransmitter Inhibition Suppressed During Gaming Explained by Skill Rather Than Gamer Status
Source: Front Hum Neurosci. 2020 Dec 7;14:585764. doi: 10.3389/fnhum.2020.585764 (PMC7750522; doi:10.3389/fnhum.2020.585764)

## Supplementary Materials

**MRI data acquisition.** A 3 Tesla Siemens Prisma scanner located in the university's imaging facility was used. The T1-weighted high-resolution anatomical image was acquired with the MP-RAGE sequence (160 sagittal slices, FOV = 256 mm, matrix = 256x256, TR/TE = 2300/2.91 ms, TI = 900 ms, flip angle = 9°, slice thickness = 1 mm, iPAT factor = 3). The voxel used to acquire MRS data was placed in the right hippocampus (voxel size 40 ´ 17 ´ 17 mm$^3$). The MEGA-PRESS J-editing sequence was used for GABA measurement: TR/TE = 1500/30 ms, bandwidth = 1000 Hz, 512 data points, number of measurements = 128. The sequence resulted in three spectra, one with the spectrally selective editing pulse centered at 1.9 ppm (edit-on), the other with the pulse centered at 7.5 ppm (edit-off) in an interleaved fashion, and their difference. The difference spectrum contains GABA peaks near 3.0 ppm. Each voxel measurement began with the FASTMAP shimming method twice (Gruetter, 1993; Gruetter & Tkáč, 2000) in order to automatically perform first and second ordering shimming. This was followed by manual shimming if FASTMAP did not provide adequate shimming results. The full width at half maximum (FWHM) of the linewidths of the water peak were all below 25 Hz after these procedures. All scans were visually checked to ensure acceptable MRI quality (see Figure 1 for sample spectra).

***Voxel Placement.*** The hippocampus was identified by locating the parahippocampal gyrus in sagittal images. The amygdala and hippocampus were then identified. Axial views were used to specify the anterior hippocampus in relation to the amygdala and posterior hippocampus. The location was verified using coronal views. Care was taken to ensure that the voxel did not overlap with sinuses, ear canals or other air pockets. Voxel placement at the region of interest is demonstrated in Figure 1.

HIPPOCAMPAL INHIBITION SUPPRESSED DURING GAMING

Figure 1

*Voxel Placement at the Region of Interest and MR Spectra from MegaPress*

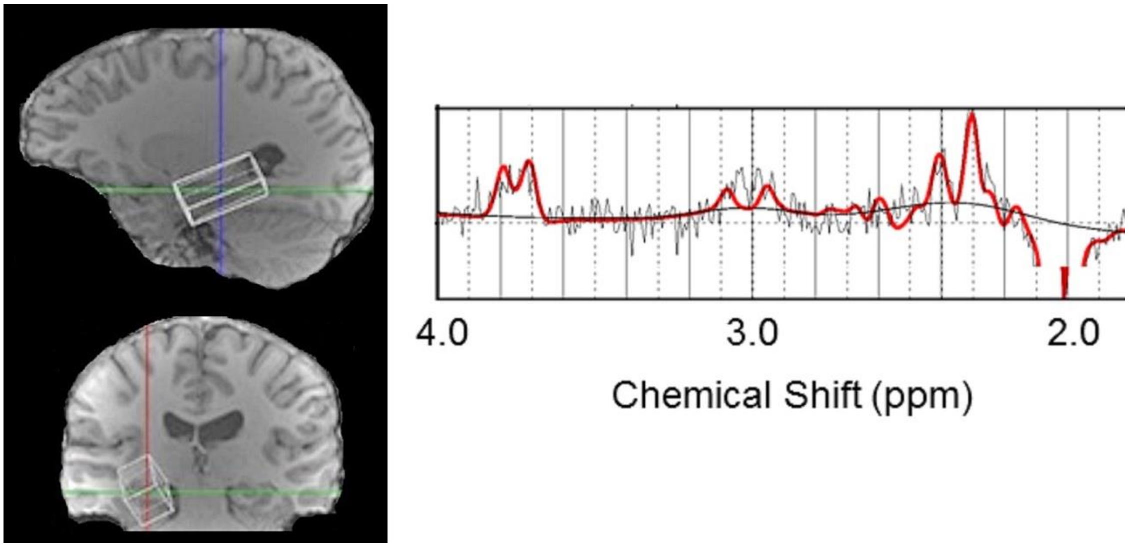

Supplement: Supplementary file 1 [file Data_Sheet_1.pdf]
